# Lessons Learned from the Japanese Encephalitis Vaccine Introduction in India That Supported the Introduction of Ivermectin–Diethylcarbamazine–Albendazole for Lymphatic Filariasis Elimination

**DOI:** 10.4269/ajtmh.21-1168

**Published:** 2022-03-15

**Authors:** Raj Shankar Ghosh, Pradeep Haldar, Julie Jacobson

**Affiliations:** ^1^ Bill & Melinda Gates Foundation, New Delhi, Delhi, India;; ^2^ Ministry of Health & Family Welfare, Government of India, New Delhi, India;; ^3^ Bridges to Development, Vashon, Washington

## Abstract

We used the introduction of the Japanese encephalitis (JE) vaccine in India as an example to understand more fully the process of introducing any new clinical product in India. We discuss the key decision-making points as well as the many activities involved in introducing a new clinical product in India’s public health program. We write from our experience in supporting the government of India to introduce new products successfully—namely, vaccines—to India’s health system. In India, the process begins with identifying the public health problem (e.g., an outbreak of JE), deciding to take action, prioritizing where action is needed, securing a supply and price of the intervention (the vaccine; in this case, the live, attenuated SA 14-14-2 vaccine), and determining how to ensure effective rollout of the intervention (the vaccination program). Reflecting on the experience of the JE vaccination program helped to inform the introduction of the triple-drug therapy of ivermectin, diethylcarbamazine, and albendazole in India as a new treatment protocol for lymphatic filariasis.

## INTRODUCTION

In India, new clinical product introduction for drugs and vaccines follows three primary pillars: 1) framing an evidence-based policy for product introduction and scale-up, 2) securing an uninterrupted supply of a safe and efficacious product the program can afford in terms of pricing, and 3) planning and executing a robust program focusing on coverage and equity.

**Figure 1.**
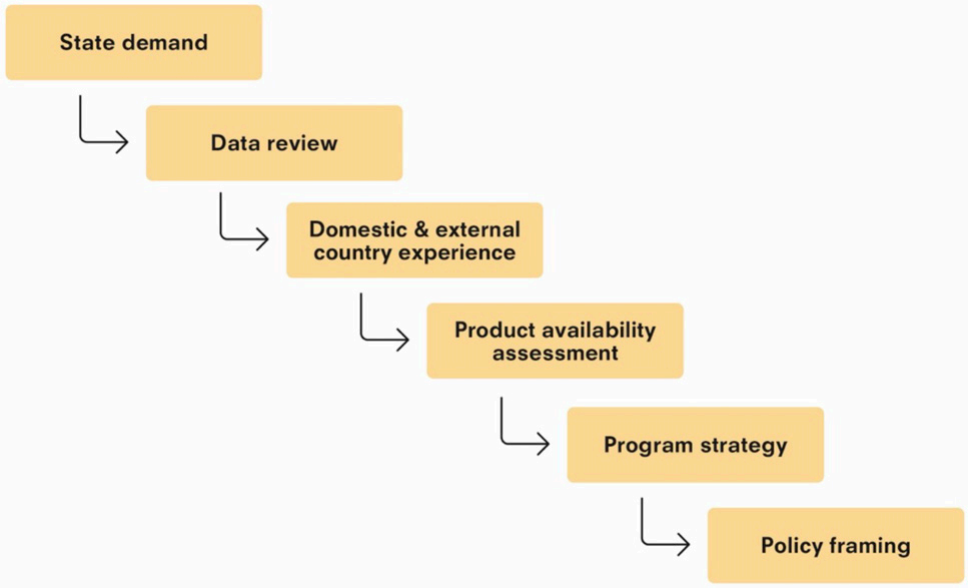
Decision to Act: From demand to framing policies for new product introduction in public health systems in India.

At the national level, the decision making is a complex, evolving process that involves multiple decisions and cannot be defined as a single decision point. The key decision points in the process can be summarized in three major steps: the decision to act, the decision to introduce an innovation and implement in the program, and the decision to continue the new program. Each of these decisions is supported by information and multiple smaller decision points by various stakeholders. The first decision—to act—sets off a series of activities before a program can be implemented. The set of activities in the process is summarized in Figure 1.

In this article, we illustrate how the steps of the process were put into action in India in 2006 for Japanese encephalitis (JE) vaccine introduction, and the lessons we learned in our roles within and supporting the Government of India throughout this process. We map out the flow of events—from the outbreak of the disease to the decision to act, to the introduction of a vaccine in the program, and, ultimately, to the decision to sustain the vaccination program based on program evaluation and vaccine supply commitment.

Reflecting on the experiences of the introduction of the JE vaccine in India later helped to inform the successful introduction of a new triple-drug therapy for lymphatic filariasis (LF), a disease that more than 430 million people in India are at risk of contracting. ^1^ India first introduced the triple-drug therapy of ivermectin, diethylcarbamazine, and albendazole (IDA) as part of its LF elimination strategy in 2018. The IDA introduction followed a similar process based on local and global evidence of the safety and efficacy of the triple-drug therapy for LF, which is discussed in other articles in this Supplement. ^2^^--^ ^7^ We highlight the lessons learned from the introduction of the JE vaccine in India in 2006.

The first clinical case of JE was reported in 1955 in Vellore (in the state of Tamil Nadu, in southern India). JE virus transmission in India was first reported in 1952 in Nagpur (in the state of Maharashtra, in western India). The first outbreak was reported almost two decades later, in 1973, in Burdwan and adjoining districts (in the state of West Bengal, in eastern India). ^8^ Since then, JE has been reported in many areas of India, particularly in the states of Uttar Pradesh (specifically in the Gorakhpur subdivision, and other districts in eastern Uttar Pradesh), Assam, and Bihar. ^9^^,^ ^10^ In the beginning, control measures were patchy, with irregular reporting and ineffective ring immunization that stretched to a few villages surrounding the village that reported a JE outbreak. This program used a domestic mouse brain-derived inactivated vaccine with moderate effectiveness (discussed later).

Historically, the reported case fatality rate of JE varied between 24% and 33%, with wide variations among states. In 2005, there was a massive outbreak of JE in eastern Uttar Pradesh, Bihar, and Assam. The government addressed the problem by launching three new program initiatives: 1) acute encephalitis syndrome surveillance, 2) a multidistrict JE vaccination campaign with an imported vaccine from China (the live, attenuated SA 14-14-2 vaccine), and 3) an information, education, and communication campaign in affected districts that followed the polio eradication initiative best practices. This required seamless coordination between the National Vector Borne Disease Control Program (NVBDCP), where JE had been addressed mainly through vector control, and the Immunization Division of the Ministry of Health and Welfare, which was responsible for executing the vaccination program. Building on a significant amount of work and lessons learned prior to the 2005 outbreak, the NVBDCP created a series of activities that supported an evidence-based response following the process, as described next.

## PROBLEM

Starting in mid-April 2005, focal outbreaks of JE were reported from the states of Uttar Pradesh, Bihar, and Assam. During the monsoon season and beyond (June–November 2005), the number of cases and deaths reported increased sharply: 5,737 persons were reported to be affected by the disease and 1,344 persons were reported to have died in the seven districts of Uttar Pradesh alone. ^11^

There were five major challenges that affected India’s national health program’s ability to deal with the 2005 outbreak: 1) the surveillance system was not robust enough to capture relevant data for effective action because of poor reporting mechanism and the limited availability of diagnostics; 2) there was a very limited supply of the domestic inactivated vaccine, and the use of this vaccine was restricted because of safety concerns; 3) the tertiary health-care infrastructure in eastern Uttar Pradesh was suboptimal and relied on only one medical college for JE management; 4) the country had multiple, concurrent health-related priorities, including polio eradication; and 5) there was no effective media outreach at the grassroots level to communicate prevailing conditions from the field effectively.

During this time, there were also various opportunities on which the JE program acted to deal with the outbreak (https://nvbdcp.gov.in/index1.php?lang=1&level=1&sublinkid=5773&lid=3693). India had two domestically developed (ELISA) diagnostic kits that tested for JE antibodies. They were available for expanding surveillance and were supported by the acute flaccid paralysis surveillance network of the WHO. These tests had started, and could be further deployed, to strengthen surveillance and provide strong evidence for decision making. India had and still has a National Technical Advisory Group on Immunization (NTAGI) constituted by the Government with local experts and international experts on invitation who were available to review scientific and programmatic evidence to recommend vaccination policies to the government. At the time of the outbreak, widespread media coverage had created strong electoral pressure on the Indian government in support of introducing large-scale vaccination programs for JE. The former Program for Appropriate Technology in Health (PATH) had recently begun providing technical assistance for JE vaccine introductions in India and other South Asian countries through a project with funding from the Bill & Melinda Gates Foundation. Early support from this project helped provide experience. In particular, in one state in India, Andhra Pradesh had shown that vaccines can impact the control of a JE outbreak. All this was happening when a relatively new vaccine—the live, attenuated SA14-14-2 JE vaccine—was available in larger quantities from China than the former inactivated mouse brain-derived vaccine. The SA 14-14-2 vaccine had been used in a few countries, including India’s neighbor Nepal, increasing the evidence in support of its broader use.

## PRIORITIZATION

The Government of India prioritized JE vaccination as the only effective public health tool for controlling the JE outbreak, based on evidence of the effectiveness of the JE vaccine in controlling transmission in many countries, specifically in Nepal and in India’s own state of Andhra Pradesh.

The NVBDCP of India and the Indian Council of Medical Research IMCR provided epidemiological and serological data to help prioritize the districts in which the intervention should be used. Public demand for vaccines both locally and at national and state levels pushed JE vaccination ahead of other public health programs as a priority for the government.

## POLICY

The policy for JE vaccination by the Ministry of Health and Family Welfare, Government of India, was created based on available evidence. As shown in Figure 1, several pieces needed to be in place to supply the information required for this policy decision. First, there was a full review of the epidemiological and serological evidence. In parallel, the evidence on the safety, efficacy, and supply of available products was compiled and reviewed for systems compatibility and affordability, both of which are discussed later. Last, the vaccination experiences of other countries and their lessons learned were taken into account. Thus, a series of events took place to gather the evidence to support a recommendation and policy decision.

The health ministry convened a scientific group to explore the potential of large-scale JE vaccination campaigns in JE-endemic districts, and the options for vaccine availability. This was done as a part of the NTAGI recommendation process. Existing JE control programs in India were reviewed. It was recognized that the JE control program run by the NVBDCP relied primarily on vector control methods, ^12^ which—over a 30-year period—had not controlled JE successfully, as recurring annual outbreaks were well established. The lack of progress in controlling these outbreaks, together with increased state experiences with vaccines and knowledge of the availability of a safe and efficacious vaccine on the horizon, led to a change in strategy. Lessons learned from countries such as Japan, Thailand, and China led the NVBDCP to consider mass immunization of the population as an effective tool for controlling JE in India.

Previous attempts in India to vaccinate children against JE were also reviewed and found to be restricted because of the inadequate availability of a JE vaccine. Vaccination campaigns had thus been limited to small, focal geographic areas, where—given the complexity of a multidose immunization schedule—there still were often insufficient doses for individuals to complete the immunization series. Although dramatic reductions in JE caseloads had been observed immediately after vaccination campaigns in these geographic areas, the lack of a sustained vaccination program limited any major long-term impacts in controlling the disease outside of focused areas in Tamil Nadu and Andhra Pradesh. However, the results in these states were very important, and the states remained key supporters of JE immunization.

With this background prepared, the policy decision proceeded, involving this three-step pathway:

1. Collation of evidence and presentation to the NTAGI.
2. Review of the evidence and recommendation by the NTAGI on introducing the JE vaccine in a national expanded program for immunization.
3. Review of the NTAGI recommendation by an inter-ministerial body (the Expenditure Finance Committee) constituted by the Government of India for budget and program approvals. This was the final approval for the introduction of the JE vaccine in the Expanded Program on Immunization).

**Figure 2.**
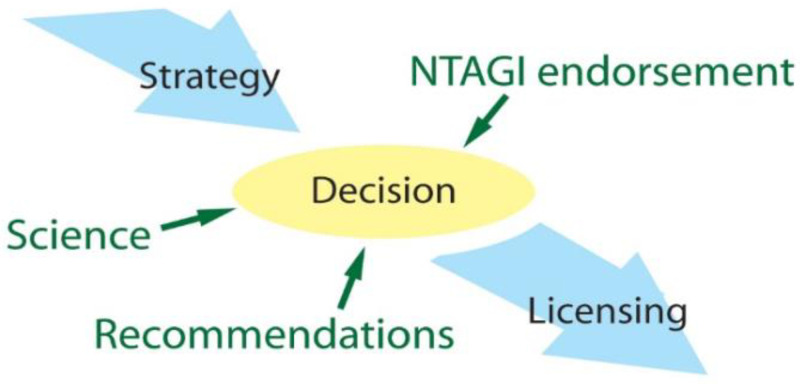
Inputs into the decision to introduce the Japanese encephalitis vaccine. NTAGI = National Technical Advisory Group on Immunisation.

After the review of all the historical experience and compiled data, the committee strongly recommended JE vaccination as the only effective tool for long-term JE control. This consensus, supported by several years of preparatory work (Figure 2), allowed for informed decision making and resulted in the ultimate decision to introduce the vaccine.

## PRODUCT

As soon as the Ministry of Health and Family Welfare decided to act, the search for an effective vaccine was initiated. It was estimated that at least 12 to 15 million doses of a JE vaccine would be required to conduct a meaningful campaign. The five essential criteria for vaccine selection were safety, efficacy, affordability, supply stability, and program compliance.

There were two vaccines available for use: a mouse brain-derived inactivated vaccine and a live, attenuated vaccine, which was the SA 14-14-2 vaccine available from Chengdu Institute of Biological Products, China National Biotech Group at Chengdu, China. The inactivated mouse brain vaccine was not able to meet the criteria for selection. The WHO had raised recent concerns regarding the safety of inactivated mouse brain-derived vaccines. The available supply of the vaccine was also only a few 100,000 doses, whereas millions were required. In addition, it was an expensive vaccine—not one that was affordable for a large campaign. Last, the vaccine was a two-dose primary series with boosters required, which raised compliance and feasibility issues. Therefore, very early in policy decision making, the inactivated mouse brain-derived vaccine was ruled out as a choice, primarily because of safety and supply concerns.

Data were then reviewed on the live, attenuated SA 14-14-2 vaccine, and it was chosen for the India program based on the advantages over the inactivated vaccine. The first was vaccine efficacy. A single dose of SA 14-14-2 has high efficacy (the vaccine is more than 80%–90% efficacious after one dose and more than 98% after two doses). ^13^ It was also recognized as the vaccine was to be used in campaigns that multiple doses increases the complexity and cost of the program. The Government of India believed there would be better compliance and feasibility with a single-dose regimen compared with the multidose regimen of the mouse brain-derived vaccine, making SA 14-14-2 a better choice for large campaigns. The vaccine had an excellent safety record, with no severe adverse events reported after immunization of more than 200 million children in China. The WHO’s Global Advisory Committee on Vaccine Safety acknowledged the excellent safety and efficacy profile of the SA 14-14-2 vaccine, with only minor recommendations for future studies. ^13^ The price of the vaccine was also favorable. The SA 14-14-2 vaccine from China was approximately 15 times less expensive than the mouse brain-derived vaccine, resulting in lower vaccine and program costs, which are discussed later. Most importantly, the manufacturer of SA 14-14-2 committed to an uninterrupted supply of the vaccine through local Indian vendors for a multiyear period based on the projection that the Government of India provided.

International experience and supporting documents contributed to the policy-level decision to initiate the JE vaccination program in India with the SA 14-14-2 vaccine. The key documents that supported the government of India decision-making process, which validated the safety and efficacy of the vaccine, supported the decision to introduce the vaccine, and provided the basis for planning the implementation strategy, are summarized here:

1. The Global Advisory Committee on Vaccine Safety article ^13^
2. Articles published in *The Lancet* on the single-dose use of the SA 14-14-2 vaccine in Nepal ^14^^–^ ^16^
3. The WHO and PATH report on the third bi-regional meeting on control of JE, held in Ho Chi Minh City, Viet Nam, April 26–27, 2007 ^17^
4. A letter from the WHO representative in India
5. The PATH Advanced Immunization Management e-Learning module, with compiled resources that include other countries’ experiences with use of the JE vaccine

## PRICE

At this time, the PATH was working actively to get an affordable JE vaccine widely available to JE-endemic countries. After extensive research and expert review of potential candidate vaccines, the PATH prioritized the SA 14-14-2 vaccine because of its safety data after broad use in China and other countries such as South Korea and Nepal. The SA 14-14-2 vaccine’s apparent durable immunity after a single dose also meant that it was good for use in campaigns as a response to outbreaks. Limitations to this vaccine included a lack of availability of original data from the Chengdu Institute of Biological Products Company (CDIBP) in English, the need for the vaccine manufacturing facility to be expanded with additional international good manufacturing practice requirements, and the vaccine manufacturer’s lack of familiarity with the process of vaccine prequalification and registration outside of China. In exchange for technical and limited financial support, the PATH’s JE project negotiated a price for the JE vaccine for JE-endemic countries that was commensurate with measles vaccine pricing, with a focus on those that were eligible under the Global Alliance for Vaccines and Immunisations. The final agreement between the CDIBP and the PATH for the public-sector price of the SA 14-14-2 JE vaccine was still awaiting signature and was not yet public information in 2006. At this time, the PATH team negotiated with the CDIPB to honor the pending public-sector pricing prior to the signing of the formal agreement with the PATH, so that this price would be available to the Government of India in time to support vaccine introduction in 2006.

The Government of India’s introduction strategy was defined based on the at-risk age groups from age-specific case reporting and the high-risk areas that were mapped previously by the states with support from the PATH’s JE project. After defining the strategy, the public-sector price for the SA 14-14-2 vaccine allowed for the introduction of the vaccine into all the high-risk areas identified by the Government of India within the available national budget.

With a suitable vaccine identified, NTAGI recommendations in place, and affordable pricing negotiated, the decision to introduce the SA 14-14-2 vaccine was made. Documentation of the reviews and decisions to date, international supporting data, and NTAGI recommendations were consolidate in a single document to serve as the basis for the decision to introduce a vaccine, for immunization planning, and for the initiation of vaccine licensing and procurement processes.

Following the decision to introduce the vaccine, activities and decisions on implementation needed to be initiated. Licensing issues arose because the vaccine was a new product to India. In China, where the product is manufactured, more than 200 million children had been vaccinated with an excellent safety record, as mentioned earlier. The vaccine was also licensed in South Korea, which had experience of using the vaccine without serious adverse events. For licensure in India, however, several milestones needed to be met. A meeting between vaccine manufacturers and the officers of the Ministry of Health and Family Welfare was held, and a scientific advisory committee was formed, with the Secretary of Health and Family Welfare as chair, to oversee the vaccine licensure process. This was all done as a part of the NTAGI recommendation process. As it did with other pharmaceutical products, the Indian Council of Medical Research took the lead in advising the Drugs Controller General of India (DCGI) on the process for registering this vaccine for marketing. A full dossier was submitted to the DCGI. The dossier’s annex included a trip report to the vaccine manufacturing plant in Chengdu, China, and provided additional insights on the DCGI’s study of locally available reports and inspection of the facility. The dossier and the trip report were reviewed by the NTAGI. After full consideration, the Indian Council of Medical Research recommended the licensure of the SA 14-14-2 live, attenuated JE vaccine manufactured by the CDIBP, as discussed in the next section.

## PROCUREMENT

A public-sector, government-owned entity, HLL Lifecare Limited (HLL; formerly Hindustan Latex Ltd.), which had experience procuring products from China for its programs, was assigned the task of importing the vaccine from China. HLL negotiated for the agreement to act as agents of the vaccine’s manufacturer, the CDIBP, and procure the vaccine. The choice of HLL for procurement of the vaccine presented several challenges. HLL is a public-sector, government-owned entity responsible primarily for the procurement and distribution of condoms, and in 2006 had no experience in the licensing or importation of vaccines. In addition, the World Bank was auditing its existing funding to the government of India as part of a larger investigation, and until the audit was complete, it was not possible to use World Bank funding to purchase the vaccine. ^18^ In light of this situation, the JE vaccination program required direct allocation from the Government of India budget to cover the vaccine costs. HLL required technical support as well as support from the PATH for cross-cultural communication with the CDIBP because of the significant differences between India and China in cultural communication styles.

For timely implementation of the JE vaccination campaign in Uttar Pradesh on the target start date of May 15, 2006, HLL navigated the procurement of the vaccine, oversaw the logistics of importation, and placed a final order with the CDIBP. This process was conducted with unprecedented speed, and the vaccine was imported in time.

## PLANNING

Four major challenges were identified and taken into account when planning the JE vaccination program: 1) accessing some of the most hard-to-reach areas of the country, including villages isolated by rivers and challenging mountain terrains; 2) reaching very high coverage for impact, especially in some areas where the health system was weak and routine immunization rates were historically very low; 3) establishing a robust vaccine safety surveillance system to capture any adverse events following immunization (AEFI) for a vaccine that had been only sparsely used in the country before; and 4) sensitizing the media on the use of a foreign, new vaccine that did not go through the mandatory bridging studies in India before use in programs.

### Accessing hard-to-reach areas.

Program managers at every level, from the Central Immunization Division down to the Block Primary Health Care Centers (known as “blocks”), relied heavily on lessons learned from their Pulse Polio vaccination campaigns. Three specific learnings from those campaigns—the three Ms—were adopted: microplanning, mobilization of community-based partners, and robust monitoring of the program.

Medical officers from the WHO’s National Polio Surveillance Project and program officers of the United Nations Children’s Fund (UNICEF) worked closely with the PATH’s JE project team to support the local government of India functionaries in training and supporting health workers to develop tailor-made micro-plans that addressed local challenges of terrain, human resources, and supply issues. Every vaccination booth had a micro-plan, which helped ensure effective implementation of the campaign.

### Reaching high coverage.

UNICEF worked closely with the Government of India’s media division to develop easy-to-understand information, education, and communication materials in local languages for effective outreach to local communities. In addition, a unique strategy was adopted that was in line with election campaigning: local people’s representatives and government officials spoke about the vaccination campaign from makeshift podiums in rural heartlands of program districts. Given the festive mood around these events, they were large gatherings, with people coming from distant villages to hear about the program. Other social mobilization campaigns included appeals from local chapters of professional bodies of medical organizations such as the Indian Medical Association. Social welfare officers in some blocks of West Bengal also put in place folksong teams from the information, education, and communication division to compose and present songs and plays in local languages to increase awareness of JE and JE vaccination.

### Establishing vaccine safety surveillance.

The Government of India placed a very high priority on surveillance of adverse events following JE vaccination. The vaccine had been used only sparsely, in one state, before the 2006 campaigns, and AEFI had not been well documented. Also, the AEFI surveillance program in the country was weak at that time. Furthermore, the live, attenuated SA 14-14-2 JE vaccine had not yet been prequalified by the WHO. The vaccine had also not undergone the bridging study in India for its introduction the program. Therefore, AEFI data had to be collected meticulously for the dossier for WHO prequalification.

The WHO and PATH national teams worked with their international offices to support the immunization division at the Ministry of Health and Family Welfare in drafting the first guidelines for AEFI surveillance for the JE vaccine. The document was reviewed by national agencies such as the aforementioned DCGI and Indian Council of Medical Research. The guidelines for AEFI surveillance were then disseminated to the blocks, and staff were trained in AEFI surveillance.

In addition to strengthening the AEFI system, a set of nested studies was included as the vaccine was rolled out. It included studies on safety and efficacy to evaluate the vaccine and the program’s success, and to support decision making on the continuation of the program.

## PROGRAM

In 2006, JE vaccination campaigns were launched in 11 districts of four states in India (Table 1). A total of 104 districts had been identified as JE-endemic districts in India, and a 5-year plan (2006–2010, as well as a mop-up campaign in 2011) to cover these districts had been created. These JE-endemic districts were identified by reviewing available surveillance data from the NVBDCP, epidemiological data, and serological data from the Indian Council of Medical Research. However, because of the limited supply of the JE vaccine initially, only 11 districts in four states were selected for the 2006 campaign.

**Table 1:** Coverage achieved during India’s Japanese encephalitis vaccination campaign, 2006

State	District	Start date	Target population size (age, 1–15 years)	Coverage, *n*	Coverage, %
West Bengal	Burdwan	June 18, 2006	2,190,690	1,229,404	56.12
Assam	Dibrugarh	September 7, 2006	409,611	370,653	90.49
	Sibsagar		372,356	276,487	74.25
	Assam total	–	781,967	647,140	82.76
Karnataka	Bellary	October 7, 2006	720,517	595,648	82.67
Uttar Pradesh	Gorakhpur	May 15, 2006	1,390,307	1,349,047	97.03
	Deoria		1,074,219	1,072,683	99.86
	Kushinagar		1,095,877	1,085,055	99.01
	Maharajganj		776,500	806,986	103.93
	Lakhimpur Kheri		1,183,481	1,218,364	102.95
	Siddharthnagar	May 22, 2006	775,934	792,944	102.19
	Sant Kabir Nagar		542,062	511,417	94.35
	Uttar Pradesh total	–	6,838,380	6,836,496	99.97
India total	–	–	10,531,554	9,308,688	88.39

The strategy was to conduct a one-time campaign targeting 1- to 15-year-olds, which was to be followed by integration of the program into India’s Universal Immunization Program. The program focused on 5 key components. The first component included the development of program guidelines in planning, training, AEFI, biological waste disposal, and communications. The second component included training. The strategy adopted was building capacity by training “national trainers,” who in turn would train “state trainers,” who would then train “district trainers.” District-level trainers would train health workers across program districts for the campaign. The training module was developed by Ministry of Health and Family Welfare and it included multiple aspects of a new vaccine launch—vaccine administration techniques, to monitoring AEFI, to communications and medical waste management. In addition, district-level members of professional bodies such as the Indian Association of Paediatricians and the Indian Medical Association were trained. A separate training and orientation of media personnel was conducted nationally to raise awareness.

The third component consisted of planning for the vaccine and logistics supply chain: This plan built on the experiences of the Expanded Program on Immunization and Pulse Polio programs. The vaccines were flown by the CDIBP from Chengdu to Delhi. HLL received the shipment at the airport and managed the customs clearance process. Vaccine samples were sent to the Central Drugs Laboratory at Kasauli for inspection of vaccine potency and quality according to WHO technical recommendations, ^19^ and the vaccine was stored at the Central Medical Stores Depot in Karnal. After the samples passed inspection, the vaccines were released and sent to other regional store depots at various points in India. From the regional depots, the vaccines were sent by cold chain vans or in cold boxes to each state’s vaccine stores, then to districts, to blocks, and, finally, the last cold chain points.

The fourth component was to develop micro-plans for local adaptation of the national guidelines at the block level. The fifth, and last, component was to set up a district task force. In line with National Polio Surveillance Project guidelines, a district task force was created in each program district to monitor functioning of the AEFI unit, the communications unit, and the cold chain units in the block. The district task force would also oversee interdepartmental coordination for smooth execution of the program.

It was also decided that there would be three tiers of monitoring and evaluation of the program. The first tier was to be overseen by the district task force and would comprise district-level monitors from medical colleges and various government departments and partners. The second tier involved concurrent monitoring of the program by the WHO and UNICEF. The third tier consisted of coverage evaluation surveys that would be conducted at intervals to cross-check reported coverage data.

The first coverage evaluation survey was conducted in 2008. The survey showed a negligible difference between reported and evaluated coverage in Karnataka and Maharashtra, moderate (10%) variation in West Bengal, and wide variation in Uttar Pradesh. In 2006, coverage in the Burdwan District, West Bengal, was particularly low, as seen in Table 1, as a result of misreporting in the press of deaths related to the vaccine during the campaign. A strong response and investigation by the government with an independent review was put into place, but not before the reporting had caused significant issues with coverage. With social media and reporting available online, repercussions of the inaccurate reports had an impact around the world that had to be managed. The Indian Academy of Pediatricians responded to the inaccurate negative communications by spreading positive messaging in the district, which helped to restart the program. This experience shows that crisis communications planning and response are essential elements of the introduction of any new intervention, especially with the rapid dissemination of information, be it accurate or not, in the modern era.

**Figure 3.**
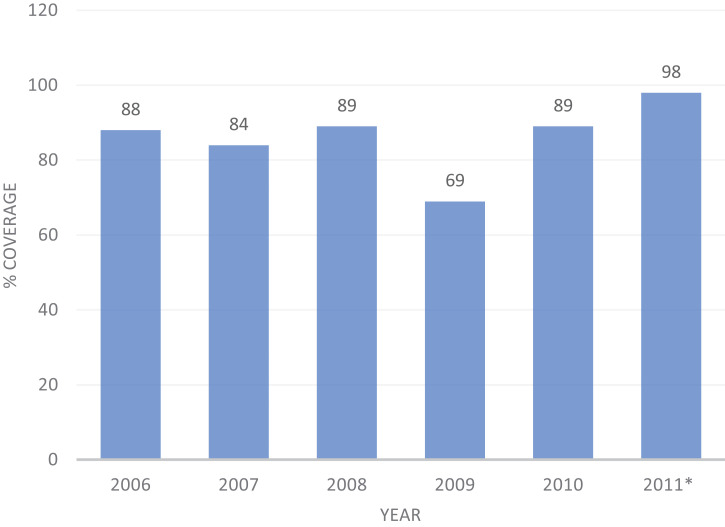
Coverage achieved during India’s Japanese encephalitis vaccination campaigns, 2006 to 2011. *2011 value includes mop-up rounds at the beginning of the year.

Overall, the first phase of the JE vaccination campaign in India (2006–2010, as well as a mop-up campaign in 2011) covered 118 districts, with an average reported coverage rate of 82% (range, 69–98%) (Table 2, Figure 3).

**Table 2:** Summary of coverage achieved during India’s Japanese encephalitis vaccination campaign, 2006 to 2011

Year	Districts covered by campaigns (through 2010), *n*	Campaign coverage, %
2006	11	88
2007	27	84
2008	22	89
2009	30	69
2010	19	89
2011*	9	98
Total	118	82

*The mop-up campaign of 2010.

## DISCUSSION

The national-level decision to introduce a new clinical product in Andhra Pradesh, India, was actually a series of decisions, each of which can become a stopping point if sufficient data and evidence are not available to support the process. A robust set of activities is required to support decision making and planning. Close partnership and collaboration among all stakeholders within the government and their supporting partners, based on several years of work, made the JE vaccine introduction process move swiftly, reaching millions of children in record time. The JE program—specifically the campaign that began in 2006—was a huge accomplishment for the Government of India because it brought in and introduced a new vaccine in 9 months to cover 8.8 million children across multiple states. This program continues today, expanding to cover new areas that are at risk and new birth cohorts that are born into high-risk settings. Because JE is a vector-borne viral zoonotic infection, humans remain at risk as a result of viral transmission in animals and pervasive mosquito vectors. This immunization program will always be needed.

Although many lessons learned from the introduction of the JE vaccine have benefited the successful introduction of the IDA protocol for LF in India, if all goes to plan, the LF program will not always be needed in the way the JE program is needed, because the program’s target is elimination of LF as a public health problem and, ultimately, the elimination of transmission. The new introduction of the IDA treatment into the LF program will accelerate this achievement. Tools developed during the JE vaccine program introduction through the new vaccine introduction process helped to facilitate the introduction, scale-up, and sustainability of the IDA program in India based on available evidence. In the same way that the JE experience helped to shape and expedite the process of introducing IDA in India, so, too, did the infrastructure, personnel, and lessons learned from the polio program help shape the introduction and rollout of the JE vaccine. Many lessons and tools, including the 3 Ms—microplanning, mobilization of community-based partners, and robust monitoring of the program—were borrowed from the successful polio program and made the relatively quick rollout of the JE vaccine possible.

## CONCLUSION

The rapid introduction of the JE vaccine in India followed years of preparatory work to support evidence-based decision making. Strong partnerships enabled quick and decisive action, which was also supported by data and tools to be successful. The key lessons learned from the introduction of the JE vaccine program in India are as follows.

First, political will stimulated through local demand and linked to data is powerful and leads to strong national ownership and, ultimately, sustainability. Political commitment at the national and state levels is necessary to carry out ambitious timelines and achieve delivery targets successfully. During the initial JE vaccination campaign, multiple barriers were overcome as a result of this strong commitment to introducing the vaccine prior to the next JE season.

Second, the ability to implement challenging health programs successfully is different now than in the past. Most prominently, the investment in polio has had an impact well beyond polio. The programmatic infrastructure put in place through the polio eradication efforts has resulted in personnel with significant programmatic experience, and processes that can be adapted to additional vaccination efforts and beyond. Without this experience, the JE campaigns would never have been completed in the incredible timelines that the Government of India was able to meet. In addition to facilitating the training and planning process, systems and forms for monitoring and evaluating the JE program were modified from the polio program, which allowed daily programmatic review to address gaps and improve program quality.

Third, decision making at the country level is not a single step. The process of introducing a new vaccine or intervention has multiple decision-making points, each of which can be rate limiting if the proper preparations are not completed and there are insufficient data and tools available to support programmatic success.

Fourth, building on an existing product introduction experience that demonstrates a clear pathway helps other programs avoid delays and anticipate next steps and potential issues.

Fifth, programs are best managed locally. Like the polio vaccination programs, the JE vaccination was managed locally under the guidance of the district task force chaired by the district magistrate. This enabled quick decisions, rapid resolution of problems, interdepartmental coordination, and an effective use of all local resources.

Sixth, and last, in this information age, programs are susceptible to new levels of misinformation and rumor spreading. This misinformation can have not only local implications, but also national and international ramifications. This issue is particularly important for campaigns that require broad participation over a short period of time. As new programs are implemented, communication plans that include local and international crisis communication responses should be considered. Crisis communications and media management require significant cultural context and understanding. Given widespread exposure through the Internet to unfiltered and substandard media sources, special plans for international communications and media management need to be considered. The impact of misinformation on the JE vaccination program is clearly apparent from the coverage data. In addition, misinformation also made it to the Internet, which amplified the impact severalfold. The management of this situation was handled well at the national level; however, the corrections to wrongly reported information did not make it to the Internet or to the international press, which has had broader ramifications.

These lessons, and the experience of the government of India’s national team and partners, provide valuable insights into how to bring new innovations to programs at scale. These lessons should help us bring new tools and approaches to programs to increase impact and improve health and well-being. Many of these lessons were drawn upon in support of the activities and studies around the introduction of IDA into the India’s LF Elimination Program, which is discussed further discussed in this Supplement. ^14^
